# Is children’s weight a public health or a private family issue? A qualitative analysis of online discussion about National Child Measurement Programme feedback in England

**DOI:** 10.1186/s12889-018-6214-y

**Published:** 2018-11-26

**Authors:** B. E. Kovacs, F. B. Gillison, J. C. Barnett

**Affiliations:** 1 Department for Health, University of Bath, Bath, BA2 7AY UK; 2 Department of Psychology, University of Bath, Bath, UK

**Keywords:** Childhood obesity, Overweight, Childhood health, Parenting

## Abstract

**Background:**

The National Child Measurement Programme (NCMP) is a child weight monitoring system in England, taking place in the first and final years of primary school. Many local authorities consider it important to inform parents if their child is overweight, and do so by letter alongside the offer of support and advice. Such letters have been met with mixed reactions from parents, but research seeking to better understand parents’ responses is often limited by reliance on survey data and low participation rates. This study aimed to collect a broad variety of perspectives on the programme by analyzing views expressed in parent-to-parent discussions posted online.

**Methods:**

UK-based online parenting fora were used to identify discussion threads based around the NCMP between 2010 and 2017. Thirty-one discussion threads from two parent fora were identified. Thematic analysis was used to identify themes in these data.

**Results:**

The primary themes identified related to (1) the legitimacy of feedback and judgement from health professionals, (2) the relative importance of collecting population level data above individual preferences, and (3) risks versus benefits of having conversations with children about weight. Most threads adopted an ‘argument, counter-argument’ format, providing two sides to each issue raised. Information and opinions consistent with public health messages were frequently provided, such as how data are used, that feedback is intended to be helpful, and the importance of collecting national data. There was little evidence of individual parents shifting their views in response to others’ arguments.

**Conclusions:**

This study provides novel insight into peer-to-peer debates about the NCMP, including the arguments parents find convincing and acceptable for and against a national programme to weigh children and provide feedback to parents about their weight. Online fora were used as an opportunity to express criticism or distress, but also to seek advice from peers regarding concerns about whether or not to opt-out. Thus, both general issues related to the legitimacy of population screening and outcomes for individual children were of concern to parents.

## Background

Childhood overweight and obesity is a serious public health concern due to the detrimental effect of excess weight on health in childhood [30], the likelihood of weight-related poor health continuing to adulthood [17, 30, 32], and the high percentage of children who are affected [22]. According to the most recent UK data, 22.6% of children in the Reception year (aged 4–5) and 34.2% in Year 6 (aged 10–11) were overweight or obese [22]. This increase between the beginning and the end of primary school is a cause for concern, demonstrating a need for effective targeted interventions to tackle childhood excess weight, prevent deterioration in health and give children the best possibilities for a healthy life.

In England, population trends of childhood weight are monitored by the National Child Measurement Programme (NCMP; [12]). The programme has run since 2006, providing annual measurements of children in the first and final years of English primary schools. The programme has extensive reach, for example in 2016 around 95% of eligible children (approximately 1,185,811 children) were measured [23]. Prior to the measurements parents receive a letter informing them of the importance of the NCMP and providing an opportunity to opt their child out of the measurements; if they do not reply, consent is assumed. The measurements are carried out within schools, usually by school nurses. Although the NCMP is designed as a surveillance programme, in most local authorities it is also used to identify children who are above a healthy weight for their height in order to make parents aware of this and offer advice or support for weight management [15]. A template for the letter, designed to provide a clear and unambiguous message in a supportive tone is provided to local authorities by Public Health England (PHE), but there is no requirement for this to be used. The template suggests that local authorities provide information on where parents can get support or advice, and clarifies that it is not intended that parents discuss the letter or its content with their child unless they choose to do so. More recent templates have been more explicit in this recommended statement (e.g., the template for 2017/2018 school year includes the statement; “The results are sent to you, so the decision of whether to talk to your child about them is entirely yours”; letters can be viewed on line at; https://www.gov.uk/government/publications/national-child-measurement-programme-operational-guidance.

The action of sharing information about children’s weight status with their parents is common to many countries (e.g., the Netherlands, and USA) in addition to the UK, and reflects both public health professionals’ beliefs that parents should be informed about the health risks of their child, and the recognition that parents have a crucial influence on the weight of their children [33]. However, such weight feedback letters and the NCMP as a whole have been met with mixed reactions from parents; some research suggests that the letter is effective in raising awareness of childhood overweight [4, 28] and that most parents agree with the feedback and find it helpful (Mooney et al. [20]), while other research reports parents feeling angry and distressed as a result, sometimes to the extent of involving the media in their complaints. However, even among those parents who report forming intentions to change their child or family’s lifestyle after learning their child is overweight, only up to half report actually doing so [20, 26].

Qualitative research investigating parents’ experiences of receiving feedback that their child is overweight provides some insight into the reasons why many react against this feedback. This includes believing that BMI is an inappropriate measure of weight status, that measurement risks making children aware that they are overweight and thus has the potential to harm their self-esteem and wellbeing, or that focusing on weight ignores other, more important indicators of a child’s health (e.g., activity levels, mental health; [8, 34]. Nnyanzi et al. [24] found some evidence of the dynamic nature of parents’ responses, where parents may initially feel shock and disgust, but later become more accepting, starting to discuss their child’s weight with friends and/or seek support. Conversely, parents of healthy-weight children report responses such as feeling relieved, congratulating themselves or feeling superior to others [24].

A further consideration that emerges is that health professionals and parents may have different understandings of, and priorities in relation to children’s health and wellbeing. Professionals are typically most concerned with long-term disease prevention, and in response target individuals to change their behavior to resolve/reverse energy imbalance (i.e., as has been termed a medicalized view) [24]. Whereas parents themselves typically prioritize children’s current mental health and wellbeing above future possible health states, and report the influence of the wider environment on childhood obesity (e.g., schools, media) that they feel should shoulder some of the responsibility for change (e.g., [8, 34]). This may account for some of the antagonism that parents express. The aim of the present study was to provide greater insight into the setting in which children’s weight-feedback is delivered, by exploring the discussions parents choose to have among themselves about the NCMP, unprompted by researchers or health professionals. To facilitate this, we drew our data from parent-initiated discussions on online parenting fora. Such fora provide space for people to express their spontaneous views on topics they themselves find relevant as they occur [31], and provide access to views that are less subject to the influence of social desirability [13], typically resulting in individuals revealing more open and honest opinions and attitudes than they do face-to-face [5]. As such, we sought to conduct an ecologically valid exploration of responses to the provision of feedback on a child’s weight through the English NCMP within parent-generated content of internet fora. Two specific objectives were pursued: (1) to explore the reasons why parents initiate and participate in online discussions about the NCMP and (2) to characterize parents’ attempts to influence or advise other parents through parent fora.

## Method

### Design and data sources

Online interactions in parent discussion fora were identified via Google search, using the search term ‘UK online parent forum’. The search and analysis were conducted in August 2016, and updated in January 2018 with any new data available from fora until the end of 2017. Fora identified in the first two pages of search hits were used as a means of limiting online data to a manageable size [27]. Relevant discussions within identified parent fora were identified using the search function, using the search terms ‘National Child Measurement Programme’ or ‘NCMP’. To ensure a representative, yet manageable amount of data, the first five pages of results for each forum were captured.

Inclusion criteria:

- Discussion threads focused on the NCMP as the primary topic (as the NCMP only takes place in the UK, non UK-based fora were excluded).
- Data from 2010 to 2017. The NCMP started in 2006, but a later start date was selected to ensure that perspectives related to a well-established programme, when standardized letters were available from Public Health England, and when local authorities had experience in running the programme.
- To retain the focus on mainstream experiences of the NCMP, fora focusing on specific sub-groups of parents (e.g. single parents’ issues) or children (e.g. with a particular disorder) were excluded.

### Ethical considerations

In line with previous online research [19, 31], consent for analysis of online data was assumed from the act of posting onto a public forum. Anonymity was assured by omitting the usernames of forum members [1, 31], and shortening quotes to reduce traceability [3]. Ethical approval was obtained from the University of Bath, Department for Health Research Ethics Committee (REACH, EP 15/16248).

### Analysis

A thematic analysis was used to explore parents’ written comments [2]. All comments were first read and coded in detail by the first author (BK) until reaching saturation in the appearance of topics raised. Comments were annotated as positive (in support of the NCMP), negative (against the NCMP) or neutral. The initial posts and the comments varied greatly in length and often contained more than one code, and therefore contributed to multiple themes. The first author then gathered the most frequently occurring codes into larger, emerging themes and discussed the justification and allocation of these with the second author (FG). Double coding was not conducted in recognition that qualitative research is inherently subjective [38], but the second author was involved as a critical friend to challenge assumptions, encourage reflection and explore alternative explanations in the coding and clustering of codes. The overall structure of the most prominent themes and relationships between them was then developed collaboratively.

## Results

### Data set

Only two UK parents’ fora were identified to provide data for this study in which posts about the NCMP were found; mumsnet.co.uk and netmums.co.uk. Mumsnet was established by a mother in 2000 and is now the UK’s largest parent network providing advice and support from parents to parents, with over 19 million visits every month. Netmums, also founded in 2000, has 1.7 million members, and around eight million users every month. The majority of members on these two forums are mothers, although no demographic characteristics were available to confirm information about people posting comments.

**Fig. 1:**
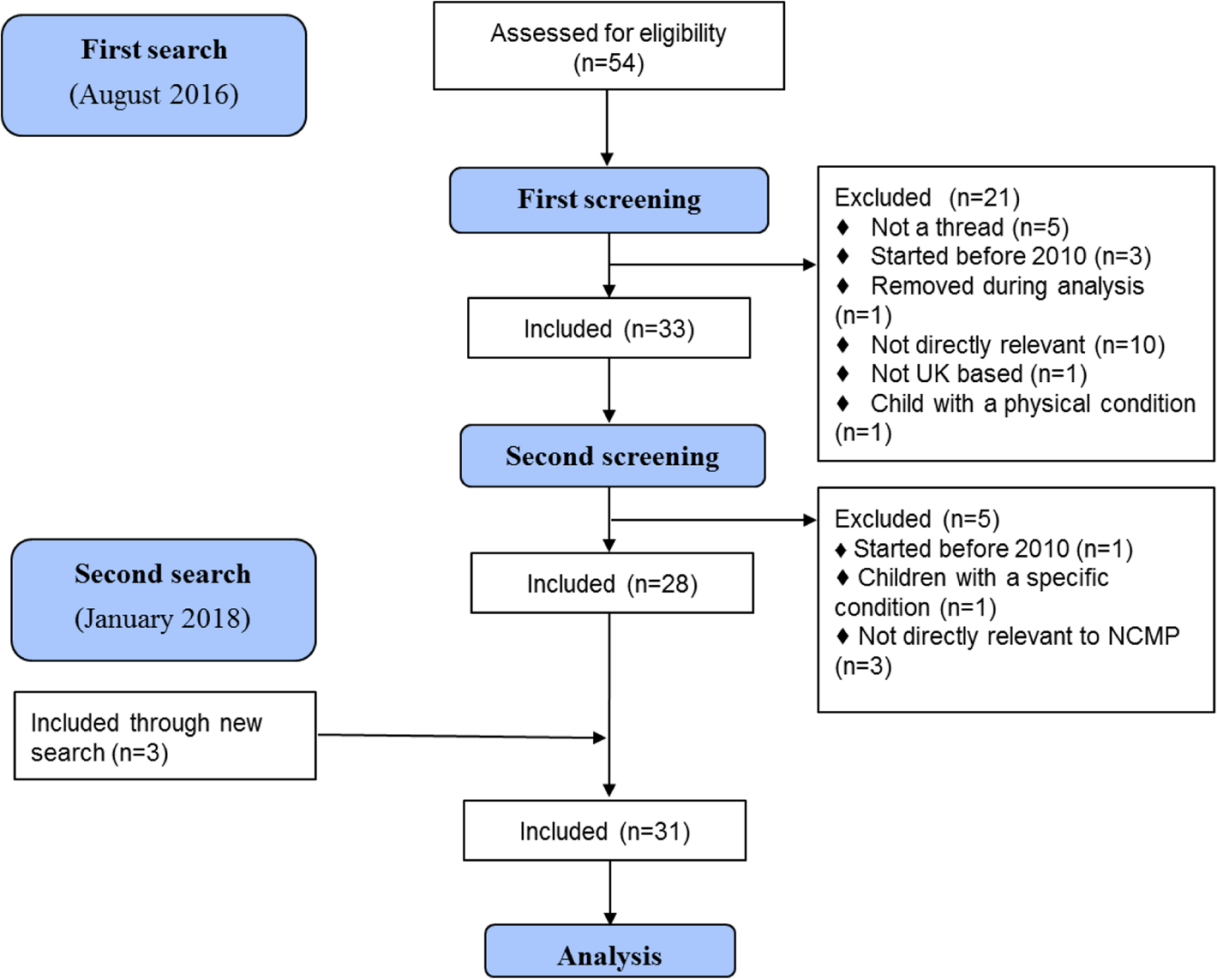
Flow chart of excluded and included discussion threads for analysis

In the initial screening process, 57 threads were identified of which 21 were excluded in the first, and five in the second round of screening as they did not meeting the inclusion criteria. Overall 31 threads were included for analysis (Fig. 1). Only three threads were identified by the second search in 2018. Thirteen threads were excluded as they were not relevant to the research question, five as the comment did not lead to an interactive thread of posts, four as they were initiated before 2010, and others as they were removed from the forum during the analysis phase (*n* = 1), was based outside the UK (*n* = 1) or related to children with specific health conditions (*n* = 2). Between four and eight discussion threads were captured each year between 2010 and 2014, two in both 2015 and 2016, while none were initiated in 2017; the greatest number of comments was recorded in 2013.

### Thematic analysis

In 19 of the 31 cases, threads were initiated by parents angry or upset with the programme, appearing to do so either to hear about others’ experiences or voice their frustrations. Some of the threads started before the NCMP was going to take place, discussing whether parents should/will opt their children out of the measurements, while other threads discussed parents’ reactions to their child’s result in the feedback letter. Eleven of the 31 threads started with a neutral tone about the NCMP, either asking a question or reporting worry upon receiving an overweight result and asking for advice without complaining. One thread was initiated with positive views about the NCMP (see Table 1 for topics of initial posts). While all threads attracted parents with similar views to contribute to the discussion (effectively endorsing the view of the person initiating the discussion), in almost all cases parents providing the alternative/opposite viewpoint got involved too. Parents sometimes challenged each other in a supportive manner (i.e., providing the voice of reason, and a rationale for why the programme could be useful), but at other times were more judgmental (i.e., that some parents needed a ‘wake-up call’). The discussions rarely progressed in a linear fashion leading to a particular conclusion (i.e., one parent/set of parents being persuaded to take another perspective); rather, the thread exemplified a range of contrasting views and claims. There was only one exception in 2016 where the parent initially posting negative comments took others’ advice that her/his son did look overweight based on a recent picture, and stated that she/he intended to make changes to his lifestyle.

**Table 1:** Initial topic of posts of included threads across years

Topic of initial post that started the discussion	2010	2011	2012	2013	2014	2015	2016
Parental consent versus opting child out (withdrawal)	x		x	x	x		
Validity of BMI as a measure of body fat	x						x
Disagreement with weight result		x		x	x		x
Concern with singling children out		x					
Criticism of the content of the letter		x					
Disagreement with running the NCMP		x	x	x	x		
Worry over the psychological effects on children (labelling children as obese)			x	x		x	
Validity of BMI for tall children					x		
Information seeking about NCMP					x	x	

Three main themes were identified; (1) Sources of legitimate feedback, (2) Intrusion versus intervention, and (3) Weight obsession versus weight discussion (Table 2). To reflect the argument - counter-argument style of the online discussions, the results will be structured by first presenting critical views followed by the contrasting positions posted.

**Table 2:** The structure of the main themes, their sub-themes and the codes that make up the sub-themes

Main theme	Sub-theme	Code
1) Sources of legitimate feedback	‘Parents know what is best for their child and do not need outside input’	Result is wrong (child is not overweight)
		External input in inappropriate/unnecessary
		Feelings (shock, fury, upset, angry, annoyed)
		Parenting failure, perceiving being told off
		More factors need to be considered (family background, lifestyle, puberty)
		Interventions should target other aspects of children’s life (school, neighbourhood)
		Parent identity
	Some parents need a reminder	Feedback letter is gentle/friendly reminder
		Overweight is perceived as the new normal in children, it became harder to notice a weight problem
		Letter contains useful advice and weight loss programmes
		Parents can be blind/biased in relation to their child and need an objective opinion
		It is better to intervene early (in childhood) than struggling with weight in adulthood
		The initial emotional harm to children is less than the overall harm of living excess weight
		BMI is a reliable tool for weight, appropriate for population level
		Health or educational professional identity
2) Intrusion versus intervention	‘Nanny state’	Distrust in government collecting this data
		The state intrudes into family life
		Nanny state (the state telling people what to do)
		Concerns about anonymity and confidentiality of children’s data
		The measurements should not be done in schools (perception of schools being behind the programme)
	Evidence based policy …how can that be wrong?’	The NHS needs statistical data to plan services locally and nationally
		Treating health problems due to excess weight costs the NHS money
		Participating is important for everyone for the sample to be representative
		Overweight in childhood can lead to overweight and health problems in adulthood
		Data collecting does not link to individual children, but is used for population statistics
3) Weight obsession versus weight discussion	Unhealthy weight obsession	Over-emphasising the importance of weight is wrong
		Measurements lead to discussing weight and upset children
		Discussing weight will lead to 'complexes' or even eating disorders
		BMI charts are one-size-fits-all and idealise a slim body shape
		Emotional harm on children is worse than being overweight
		Parents will deal with real problems when they arise (i.e. childhood overweight is not real problem)
		Parents’ own history of childhood weight complexes and/or eating disorders
	Healthy weight discussion	Weight is natural part of life and discussing it will not do harm
		Parents themselves decide whether they conduct healthy discussion about weight
		Weight is not important in relation to an ideal appearance but in relation to health
		Measurements are done sensitively and results not discussed with children to avoid harm
		The BMI chart allows for a wide range of normal, and is not trying to fit different shapes into the same size

#### Theme 1: Sources of legitimate feedback

**‘Parents know what is best for their child and do not need outside input’.** Discussion threads that were started by parents objecting to the NCMP stated that any input about their child’s weight from external parties is unnecessary. This was commonly based on either the belief that the result for their child was inaccurate, or that it is not appropriate for school nurses to get involved in children’s weight management even when a child is acknowledged to be overweight. Parents used words conveying strong negative emotions such as feeling “*furious*”, “*annoyed*”, “*shocked*”, “*frustrated*”, “*pissed off*” or “*upset*” about the result or the tone of the letter. Some described the letter as “*patronising*”, and felt like they were being “*told off*” or “*told what to do*”. Among those who did not agree with the judgment of their child’s weight status, there was a commonly expressed belief that the assessment “*needs to take account of more variables than just height and weight*” (2010). Parents felt that factors such as family background, activity levels, puberty and build (“*some people really are more sturdily built, and physically denser than others*”, 2010) should be taken account of, which they as parents are aware of but external parties measuring BMI are not.

A number of parents acknowledged that their child was overweight, but felt that the letter implied that parents were solely responsible for their child being overweight, which they believed to be unfair. These parents commonly argued that interventions should target other determinants of children’s lifestyles which, they believed, had a stronger influence on their weight. For example, parents called for the school to provide cooking classes to raise awareness of nutrition, “*offering a balanced diet at lunch time*” (2010), “*encouraging competitive sports*”, “*not having cake sales”* (2010) and opposed school fetes being sponsored by fast food companies. Parents talked about these external factors as influences on their child’s weight that were outside their control, making it unjust to imply that parents should take full responsibility for tackling children’s weight.

**‘Some parents need a reminder’.** Many parents appeared to take a mediating and supporting role on the fora to temper others’ negative feelings and beliefs. For example, they suggested that the NCMP feedback letter could be seen as “*gentle guidance*” (2013), given “*in the spirit of helping*” (2016) “*to encourage healthy eating and exercise*” (2012) in families. Expressions of empathy were also made, such as observations that as the general population is becoming increasingly overweight, “*as a society our view of normal [weight] is increasingly distorted*” (2016), so “*people are getting worse at seeing a ‘healthy’ weight vs overweight*” (2014). In many cases it was not clear whether the parents providing counterarguments had themselves received a letter, or were reporting hypothetical ways in which the feedback could be interpreted. However, there were some cases of parents who had been told their child was overweight sharing their more positive interpretations of the process; “*the letter also mentions activities provided by the council (…) to help achieve a healthy weight*.” (2010).

More critical and judgmental comments about parents of overweight children included arguments that some parents “*can have a blindness to their child’s real size*” (2013) and should be taking more responsibility for a child’s weight; “*someone needs to tell parents there is a problem and make the parents address it*” (2013). These arguments often received counter-replies from other parents arguing that weight is only “*one measure of health, not the only measure of health*” (2013), and that parents know their children’s specific needs and are well-equipped to meet them. Another response from a parent objecting to the programme suggested some parents engaged in ‘othering’; that is, the parent stated that they “*don’t have a problem with them doing the health checks*” and some *other* parents surely need the letter to notice their child is overweight, but they “*just didn’t feel it was necessary*” for *their* child (2013).

Comments posted by parents in support of the NCMP referred in a somewhat derogatory fashion to the ‘*I know best’* category of parents who would not listen to independent expert opinion. There was a feeling running through the counterarguments that “*the best time to intervene is as soon as possible*” as it is “*easier*” when habits are still developing and parents have more control. One mother believed that although there may be minor “*emotional harm*” on receiving the letter, children may later be grateful to their parents for helping them reach a healthy weight.

Some arguments were made by parents presenting themselves as experts, or referring to more informed clinical or scientific evidence. For example, one parent commented in defense of the use of BMI, “*BMI is a tool to indicate overweight. [It is designed] for population levels and is much more feasible than other measurements*”, and others provided clarifications in support of the programme often added they are health professionals themselves: “*I was a School Nurse for a while*” (2013). One of them even asked parents to “*try not to take it [their anger] out on the school nurses*”, as they want to help parents and the fact they carry out the measurements “*doesn’t mean we think you’re a bad parent*” (2012). It is therefore likely that some comments crossed the boundary of the personal/professional opinion.

#### Theme 2: Intrusion versus intervention

**‘Nanny state’.** In contrast to the debate in Theme 1 that predominantly focused on parents’ responses on behalf of their individual child, this theme describes comments at a more general level, predominantly in relation to whether the government should be carrying out such measurements. Many commenters felt that “*The government can mind its own business*” (2011), criticizing the NCMP as “*the nanny state making decisions on behalf of parents*” (2011). This opposition extended to concern around the use of the data: “*I’m not comfortable with the excuse of Gov[ernment] statistics*”. Parents raised concerns that the information collected was not anonymous, and might be shared with other parties. The fact that the data was collected in schools rather than within the healthcare system appeared to contribute to these concerns, the latter being considered as being more likely to manage sensitive information safely (“*If I* was *concerned, I would take them straight to the doctor*”, 2013).

**‘Evidence based policy ...how can that be wrong?’** Parents providing counter-arguments challenged views that children’s weight data is “*none of the government’s business*” by asserting that the NHS needs statistical evidence to “*plan ahead*” about what services they need to provide for people with weight-related conditions: “*If we didn’t have the childhood measurement programme we wouldn’t know that children, (…) were getting heavier*.” (2016). Defense of the NHS and its budget was brought into many counter arguments; “*Over time very overweight people cost the NHS more money*” (2014); “*The NHS cannot afford to keep firefighting, early detection and intervention is required*” (2013). Well-informed responses also mentioned evidence of the likelihood of overweight tracking through childhood and to chronic disease in adulthood. Replies from parents who were negative about the NCMP included mistrust in statistics “*statistics lie regularly based on whose (sic) controlling them*” (2013), and arguing that the NHS wastes money on this programme while there are more important issues requiring funding.

It was interesting that some parents presented a case for it being every parent’s duty to have their child weighed. One mother appealed to those who were undecided as to whether they would allow their children to take part; “*please do reconsider participating in the data collection*” as “*if bigger children aren’t measured it skews the population norms*” (2014). Replying to these claims, one of the original commenters replied they do understand this argument, but drawing on personal reasons, she will still “*go with my gut and refuse permission [for nurses to weigh her child]*” (2014).

Responding to people perceiving the collection of personal data as intrusion into family life, some commenters argued that the data is collected about the population, and while the letters concern individual children, they are standardized and do not target any family personally (“*Its all anonymous at stats level, postcodes only*”, 2013).

#### Theme 3: Weight obsession versus weight discussion

**‘Unhealthy weight obsession’.** A third topic raised in objection to the NCMP related to beliefs that measuring children wrongly over-emphasizes the importance of weight to children, increasing the risk of them developing ‘*complexes*’ and eating disorders. Parents raising this concern believed the programme reflected an “*obsession with weight*” and idealized a slim body shape that was not healthy or attainable for aesthetic rather than genuine health reasons; “*Here’s to the next generation of anorexia/bulimia epidemic!*” (2012). Some people also stated they believed this measurement implies that all children need to “*fit the parameters of some [BMI] chart*”, when in reality “*people come in all shapes and sizes*” (2013). Parents with more extreme views believed that this meant the process of measuring children is “*far more dangerous than the weight itself*”. Stories were shared of children becoming distressed at schools and were “*discussing their weights and saying who was the biggest*”, “*one of the children even cried*”, and a parent started a thread saying that her child read the letter and it made him/her become “*body conscious*”, which she perceived to be very harmful. There were also a number of parents who agreed that their child was overweight, but that they did not feel that this was a sufficient risk to health on its own to warrant action. They asserted they would “*tackle the problems when they happen*” (2012); only if “*it is causing health issues then there is cause for concern…*” (2012).

It was also apparent that many mothers were drawing on their own childhood memories when their own weight was criticized by adults, or when they had felt “*uncomfortable*” about their figure as a child. One mother admitted she “*was anorexic for 6 years*” (2012), while a commenter on another thread said she “*suffered years of mild eating disorders*” (2010). All comments referring to mothers’ own previous experiences were followed by strong views against weighing children to prevent them from “*ever having weight issues*”; ‘weight issues’ here referred clearly to the threat to psychological wellbeing of being worried about one’s weight, rather than issues related to physical health.

**‘Healthy weight discussion’.** Direct counterarguments to claims about the risk of weight ‘complexes’ and eating disorders were presented in response: “*Weight (both gain and losing) is such a natural part of life*” (2013), and “*It isn’t about image and looks. It is about health*” (2013). According to these commenters, discussing weight could be done in a healthy, non-judgmental manner, while opting out may create a feeling that “*getting weighed is something to worry about*” (2013). Parents of the opposing side of the debate replied to these by stating that they would rather have this attitude than their children thinking that their perfectly healthy body is wrong just because they “*don’t fit exactly into a scale [weight chart]*”.

In contrast to personal experience leading parents to avoid weight measurement for their children, one parent who admitted to having suffered from eating disorders felt the measurements could be positive. She was made to believe that overweight was something to be ashamed of when she was younger but explained that she would like to raise her children in the belief that weight is simply one of their characteristics, and overweight is something that can be improved by starting a healthy weight discussion.

A number of parents on the fora attempted to defend the programme by providing clarification about the way the NCMP takes place, for example that “*only the nurse can see*” children’s weight, and a professional working in the measurement team added “*information is strictly NOT shared with pupils*” (2012). Parents also reacted to complaints about the unsuitability of BMI and its perceived narrow range of healthy weight. They emphasized that the programme raises children’s awareness that “*we all have different weights and there is a wide range of normal*” (2013), and that “*BMI is a good rough starting point for a healthy lifestyle chat*” (2013).

## Discussion

This study investigated the topics raised by parents in relation to the English National Child Measurement Programme on UK parenting fora, and how they are discussed among peers. Two relevant fora were identified, providing 31 parent led threads for analysis. Almost two thirds of threads were initiated with critical comments about the programme, only one thread started in a supportive tone and the rest were neutrally seeking advice/information. Three key themes were identified in relation to the content of posts; debates about the legitimacy of the feedback relative to trusting parents to know best when it comes to their child’s weight; whether the programme is indicative of a ‘nanny state’ or justified for the good of the wider population; and whether concern with children’s weight reflects attempts to ‘conform’ to a certain size while over-emphasizing weight, or is genuinely proven to be important for their health. Past work has reported many of the objections to the NCMP that were recorded on these fora (e.g., [4, 8], Syrad et al., 2014, [26]), but through presenting both the argument and the counter-arguments within peer debates this research sheds light on the alternative, more supportive viewpoints held by other parents. Not only did parents go online to defend the programme but were active in providing reasons for others to take the letter as a friendly advice or in encouraging parents to let their children take part. Their counter-arguments variously appealed to the wellbeing of the child as well as the importance of the data and of national programmes on behalf of the nation.

The first theme captured the strength of emotion in the negative reactions that some parents had to the NCMP, in the belief that in providing weight feedback school nurse teams were inferring parents were not the best judge of their child’s health and what is good for them. This finding, and suggestions such as the need to take individual lifestyle factors into account, is consistent with past work [8, 35]. Other parents rejected the premise that they should be held responsible for trying to help their child lose excess weight, arguing that factors contributing to the obesogenic environment (such as school) played a stronger role and that parents could not fully control their child’s weight even if they wanted to. The National Institute for Health and Care Excellence (NICE) guidelines for childhood obesity prevention reflect a similar view by identifying schools and local institutions to be involved in creating healthy environments for children [21]. Several experts also draw attention to the impact of environmental factors such as food advertising on television [10], level of deprivation and the density of fast food restaurants in a neighborhood [6], family income [9] and even the prevalence of negative life events in childhood [18] on children’s weight. Thus, with the absence of acknowledgement that children’s weight is a product of multiple factors, the NCMP letter appeared to be either an additional irritant to parents, or justification to object or discount its validity.

Among parents’ counterarguments to those criticizing the NCMP were strong rebuttals of parents’ ability to make objective judgments about their own child’s weight, especially in a society where overweight and obesity are becoming normalized. These are consistent with research reporting that many parents do not recognize overweight in children in general [16, 14] or when judging their own child [25, 35]. The discussions generated by these comments demonstrated a tension between beliefs that early intervention has a better chance of success, and beliefs that being overweight at a young age is not a health risk and interventions only need to start when children reach more extreme levels of overweight. Similarly, there were other examples where parents (who may have also been health professionals) contributed clarifications and accurate evidence in relation to other technical points to the discussion; for example in defending of the use of BMI to establish weight status, and that all measurements remain anonymous and hidden from children. Thus, a second key finding of this analysis was to confirm that those on these parenting fora are being exposed to information consistent with professional advice in addition to opinion.

While the first theme focused on discussion of the rights or wrongs of the feedback in relation to a parents’ own child, the second theme reflects the debate around the legitimacy of the NCMP as a government policy in general. Comments relating to this theme often started before the NCMP took place in a parent’s local area (i.e., before parents were aware what the result would be for their child), and were initiated to inform parents’ decision making around whether a child should be withdrawn from the process in principle. These views question the legitimacy of the measurement and challenged it happening in its present form at all. Researchers have also argued that informing parents of their children’s weight is ethically controversial on the basis that there can be unintended consequences for those measured that goes beyond what can be agreed through providing consent to be weighed [37]. Parents critical of the NCMP referred to it as an example of interference of a ‘nanny state’ and an undesired intrusion into their lives. The discussions reported here extend this to show the additional perspective of mistrust about how children’s data will be used and shared; in some cases this was exacerbated by the measurements taking place in school rather than at (better trusted) health facilities.

The third theme revolved around the notion that the mere discussion of children’s body weight with or in front of children can increase their risk of weight obsession or eating disorders. This is consistent with concerns voiced through other research of the conflict that parents face in wanting their child to be healthy but wanting to maintain their self-confidence and wellbeing ([11]; Syrad et al., 2014). Uncertainty over whether or not weight is a problem for health during childhood led some to be unwilling to take preventive measures at all [36]. However, such rationales for lack of action were not universal; many parents took the categorical position that weighing children, especially in school in front of their peers was always problematic and believed it was driven only by aesthetic reasons. A characteristic of this theme was how parents drew on their own negative experiences as an overweight child in deciding against the NCMP. The current operational guidelines of the NCMP reflect parents’ concerns to some degree by suggesting that parents are not expected to discuss the feedback with their overweight child (it is their choice whether they do so) but should make necessary lifestyle changes in a relaxed manner without the child being aware of why [29]. However, at least one parent reflected on the potentially stigmatizing effect that not talking about weight could have, which could lead to be something to be ashamed of rather than a challenge for many families that is a normal part of life.

### Strengths and limitations

A key strength of this study is the use of an alternative source of data to gain insight into discussions traditionally accessed through interviews, focus groups and survey data. Using online discussion fora allowed views to be captured in a more natural situation [31] than when respondents are recruited for a study, avoiding social desirability relating to the research process [13] or the agenda of researchers. It also facilitated a potentially broader mix of participants, given the tendency of research participants in face-to-face studies to be from higher socio-economic groups, and/or to be motivated by particularly strong concerns they wish to raise. Further, by basing the study on data collected online, we were able to take advantage of people’s tendencies to express their views more spontaneously and honestly than may happen in person [5], particularly in relation to such a sensitive topic as obesity. Our sampling technique allowed us to include the salient themes emerging from the majority of threads found on parent fora about this topic over the last 8 years.

This study also has several limitations. First, the forums included in this study were primarily aimed at mothers, and so it is not clear what fathers' perspectives are on these issues. Further, the results were only obtained from parents who participate in online discussion, and because we do not have any demographic information about the group of commenters it is difficult to draw any conclusions about the representativeness of this sample of parents in receipt of NCMP letters in relation to the population as a whole. However, the large dataset and the long time interval allowed for the most robust themes to clearly emerge, somewhat increasing reliability. In order to ensure the potentially extensive dataset stayed at a manageable size the authors chose to include the parenting fora listed on the first two pages of search hits, and the first five pages when including discussion thread for analysis. This may have resulted in missing certain discussions, although this cut-off was chosen as there were rarely any discussions beyond the fifth page.

Lastly, due to the lack of information about the sample characteristics other than what they choose to share in their comments, it was not possible to make inferences about themes in relation to age, geographical area, socio-economic background, profession or their own child’s weight status and lifestyle.

### Implications and future research

Health professionals and public health teams are already aware of many reasons why parents object to the feedback they receive through the NCMP ([8]; Syrad et al., 2014), and that such views are not universal as there are also those who agree that the process is justified and potentially useful (Mooney et al., 2011; [4]). The present study adds novel insights as to how parents discuss issues around measuring children, and adds to our understanding of the basis on which parents are willing to challenge each other’s views, at least online. Online discussions were initiated both before and after children were weighed, suggesting that parents are concerned with the measurement of children in general, and not only by the judgement of their own child’s weight. While there was evidence of parents challenging and trying to persuade each other towards a different point of view, there was little evidence that parents were able to influence each other’s viewpoints through online debate. However, the fact that some parents were willing and motivated to present supportive arguments could suggest a direction for future research in engaging parents in the provision of peer support and education as an alternative to providing feedback by letter, or endorsed by professionals. The findings also suggest that there may be value in exploring whether clarification of what the children’s data will or will not be used for, and of acknowledging the effect of an obesogenic environment beyond the home, might allay some parents concerns about being judged and make the feedback more acceptable. However, there are other concerns that we do not yet have the evidence to address, such as the possibility that talking to children about their weight, and letting them know that they are overweight may be harmful to their wellbeing, as some parents believe [7].

## Conclusion

The current study has provided insight into the discussions parents have among themselves regarding the weighing and measuring of children, tapping a source of online information not previously explored in this subject area. Using parent instigated and generated data, this study reflects what parents believe to be important within this debate and how they defend their decisions on the topic. As the feedback from the NCMP in England focuses on encouraging parents to take action when a child is overweight, a better understanding of their views and which rationales they find relevant and convincing is an important part of improving parent- health professional communication.

## Abbreviations

BMI: Body mass index
NCMP: National Child Measurement Programme
NHS: National Health Service
PHE: Public Health England
